# Progress in the Treatment of HIV-Associated Lymphoma When Combined With the Antiretroviral Therapies

**DOI:** 10.3389/fonc.2021.798008

**Published:** 2022-01-13

**Authors:** Chaoyu Wang, Jun Liu, Yao Liu

**Affiliations:** Department of Hematology-Oncology, Chongqing Key Laboratory of Translational Research for Cancer Metastasis and Individualized Treatment, Chongqing University Cancer Hospital, Chongqing, China

**Keywords:** HIV, diffuse large B-cell lymphoma, Burkitt’s lymphoma, Hodgkin’s lymphoma, treatment, prognostic

## Abstract

With the wide use of combination antiretroviral therapy (cART), the life expectancy of HIV-infected individuals drastically improved. However, HIV infection and HIV-associated cancers were the most common causes of death in the HIV-infected populations. The HIV-associated cancers are divided into acquired immune deficiency syndrome (AIDS)-defining and non-AIDS-defining cancers based on the incidence among the HIV-infected patients. Among HIV-associated cancers, acquired immune deficiency syndrome-related lymphoma (ARL) is still the most common condition and the leading cause of HIV/AIDS-related deaths. Diffuse large B-cell lymphoma (DLBCL) and Burkitt’s lymphoma (BL) are the most common subtypes of the ARL. Although Hodgkin’s lymphoma (HL) is not considered as an AIDS-defining cancer, incidence of HL in HIV-infected individuals is higher than the general population. The review summarizes the new progress in the treatment of HIV-associated lymphoma.

## Introduction

According to the 1993 revised classification system for human immunodeficiency virus (HIV) infection, HIV-associated cancers are divided into acquired immune deficiency syndrome (AIDS)-defining and non-AIDS-defining cancers based on the coincidence among HIV-infected patients. Non-Hodgkin’s lymphoma (NHL), Kaposi’s sarcoma (KS), and invasive cervical cancer were considered AIDS-defining cancers. The other cancers such as lung cancer, anal cancer, Hodgkin’s lymphoma (HL), oral and pharyngeal cancers, hepatocellular carcinoma, vulvar cancer, and penile cancer were considered non-AIDS-defining cancers in patients with the HIV infection (1, 2).

Since 1996, with the wide use of combination antiretroviral therapies (cART), the incidence of malignant cancers in people living with HIV (PLWHIV) has decreased significantly. Nonetheless, AIDS-related lymphoma (ARL) is still the leading cause of HIV/AIDS-related deaths. Diffuse large B-cell lymphoma (DLBCL) and Burkitt’s lymphoma (BL) are the two main subtypes of ARL. Also, DLBCL is the most common subtype of B-cell lymphoma or NHL in HIV-negative patients (3).

### Epidemiology

A Swiss HIV cohort study (SHCS) included 12959 people living with human immunodeficiency virus from 1984 to 2006, and among them, 429 NHL cases were identified. In the study, the highest incidence of the NHL was 13.6 per 1000 person-years in 1993-1995. Since 1996, with the wide use of cART, the incidence of NHL declined to 1.8 per 1000 person-years in 2002-2006 (4, 5). Before the cART, the DLBCL and primary central nervous system lymphoma (PCNSL) were the most common cancers, with an incidence of 4.53 and 2.33 per 1000 person-years, respectively. But in 2001-2007, the incidence of the DLBCL and BL changed due to the development of cART treatment. The DLBCL and BL were the most common cancers, with an incidence of 1.20 and 0.32 per 1000 person-years, respectively (6, 7).

A retrospective analysis from India showed that the ARL accounted for 6.5% of all lymphoma subtypes. In the study, a total of 1226 lymphoma patients were retrospectively analyzed between March 2010 to August 2017 at Kidwai Cancer Institute. Among them, 80 patients (6.5%) were detected with the HIV infection, and 70 of these patients had complete clinical and pathological details. The subtypes of the ARL were 33 DLBCL (47.1%), 15 plasmablastic lymphoma (PBL) (21.4%), 12 Hodgkin’s lymphoma (HL) (17.1%), 3 Burkitt’s lymphoma (BL) (4.3%), 2 high grade B-cell lymphoma (2.9%), 2 follicular lymphoma (FL) (2.9%), 1 PCNSL (1.4%), 1 primary effusion lymphoma (PEL) (1.4%), and 1 peripheral T cell lymphoma (PTCL) (1.4%) (8). Several other studies have shown that the most common subtypes of the ARL is the DLBCL, followed by BL (9–14). Multiple factors have been shown to influence the pathogenesis of HIV-associated lymphoma. Immunosuppression and coinfection with viruses carrying oncogenic proteins, primarily HHV8, CMV and EBV, contribute to HIV lymphomagenesis, varying by lymphoma subtype (10). The review focuses on the progress in the treatment of the above-mentioned HIV-associated lymphoma.

## Front Line Treatment of ARL

### HIV-Associated DLBCL

NHL is the most common hematological cancer, in which, the DLBCL is the most common lymphoma in adults. The DLBCL accounted for 30%-40% of NHL in non-HIV infected patients, and about 45% in the ARL (15, 16). For the HIV-negative DLBCL, standard first-line chemotherapy is the combination of rituximab and CHOP (R-CHOP, rituximab plus cyclophosphamide, doxorubicin, vincristine, and prednisolone). However, some determinants influencing chemotherapy and outcome in the HIV-positive DLBCL cases are lymphoma-related factors (stage, size, localization, and IPI), HIV-related factors (CD4 cell count, viral load, infections, and cART), patients-related factors (age, ECOG, and comorbidity), and practitioner-related factors (experience in lymphoma therapy and experience in HIV therapy) (7).

In the cART, Little et al. demonstrated that the DA-EPOCH regimen had an 87% overall response rate (ORR), 74% complete response rate (CR), and 13% partial response rate (PR) in patients with HIV-associated DLBCL. Compared with the patients with the lower CD4 cell count (<100/mm^3^), the patients with the CD4 cells greater than 100/mm^3^ achieved higher CR (87% and 56%, respectively, p=0.04). After a median follow-up of 53 months, the progression-free survival (PFS) was 73%, and the overall survival (OS) was 60%. Compared with the cases with the lower CD4 cell count (<100/mm^3^), patients with CD4 cells greater than 100/mm^3^ achieved a higher OS rate (87% and 16%, respectively, p=0.001) at 53 months (17).

Coutinho et al. showed that the patients with HIV-associated DLBCL had good outcomes when were treated with rituximab plus standard chemotherapy (R-CHOP). A retrospective study included 305 DLBCL patients, of which 97 were HIV-positive patients from 2003 to 2011. The ORR of the HIV-negative group and the HIV-positive group were 84% (172/208) and 86% (84/97), respectively. Meanwhile, the CR for both groups was 73% (150/208 and 71/97). After a median follow-up of 48 months, five-year disease-free survival (DFS) was 77% (95CI, 67%-84%) and 94% (95%CI, 81%-97%) for the HIV-negative and HIV-positive patients, respectively (p=0.03). Compared with the HIV-negative patients, the five-year OS rate for the HIV-positive patients was higher [78%(95%CI, 68%-85%) vs. 64% (95%CI, 56%-70%), respectively, p=0.03]. On multivariate analysis, ECOG PS at least 2 [95%CI, 2.5 (1.211-5.231), p<0.01], and CNS involvement [95%CI, 5.2 (1.236-22.171), p=0.03] were predictive of EFS, not achieving a CR [95%CI, 11.4 (3.791-34.295), p<0.001], age at least 60 years [95%CI, 4.1(1.030-16.390), p=0.05] and at least two extranodal sites [95%CI, 3.0 (1.086-8.341), p=0.03] were predictive of OS. Hence, the choice of chemotherapy in DLBCL patients should not be influenced by the HIV status (18).

A phase 2 study assessed the role of the SC-EPOCH-RR regimen in newly diagnosed HIV-positive DLBCL. Thirty-three patients with untreated HIV-positive DLBCL patients were enrolled between June 2001 and April 2009 at the National Cancer Institute. The median age was 42 years (9-61 years), 76% (25/33) had a high age-adjusted international prognostic index (aaIPI 2-3 score), and 82% (27/33) had advanced stage (stage III/IV) of cancer. Regarding the treatment, 79% of patients (26/33) received 3 cycles, 6% of patients (2/33) received 4 cycles, and 12% of patients (4/33) received 5 cycles of the SC-EPOCH-RR regimen. The ORR, CR, and PR were 94% (31/33), 91% (30/33), and 3% (1/33), respectively. At the median follow-up of 5 years, the PFS and OS of these patients were 84% and 68%, respectively (19). Based on the above results, the SC-EPOCH-RR regimen is an important treatment strategy for the HIV-positive DLBCL cases.

AIDS-Malignancies Consortium (AMC) trial 010 was a randomized multicenter phase III clinical study, which was designed for patients with HIV-positive non-Hodgkin’s lymphoma. The study aimed to determine if the R-CHOP regimen would improve clinical outcomes in the HIV-positive aggressive B-cell lymphoma, compared with the CHOP regimen. A total of 150 patients were enrolled from the 22 participating AMC sites, and 99 patients were assigned to the R-CHOP regimen group and 51 patients were allocated to the CHOP regimen group (2:1). The DLBCL, BL, high-grade non-Hodgkin lymphoma not otherwise specified (HG NHL NOS), and other pathological types were 80 (81%), 8 (8%), 4 (4%), and 7 (7%), and 40 (78%), 6 (12%), 5 (10%), and 0 in two groups, respectively. The CRs were 57.6% for the R-CHOP regimen and 47.0% for the CHOP regimen. The progressive disease (PD) rate was greater in patients receiving the CHOP regimen alone (21.6%), compared with the patients receiving the R-CHOP regimen (8.1%). However, with a median follow-up time of 137 weeks, there were no statistically significant differences in the PFS time (45 weeks and 38 weeks, respectively, p=0.67) and the OS period (139 weeks and 110 weeks, respectively, p=0.76) between the R-CHOP regimen group and the CHOP regimen group. Therefore, the R-CHOP regimen chemotherapy only improved the CR and did not result in a higher PFS and OS time in patients with the HIV-positive DLBCL compared with the CHOP regime chemotherapy (20). However, another clinical study (21) and a pooled analysis (22) that consisted of 1546 patients from 19 prospective clinical trials showed that the R-CHOP regimen chemotherapy was associated with the higher CR rate, improved the PFS and OS. Compared with the CHOP regimen, first-line therapy with the EPOCH regimen was associated with the higher CR and significantly better OS in the HIV-positive DLBCL (22). However, there has never been direct comparison of R-CHOP vs. DA EPOCH-R. Concurrent use of the cART and rituximab in intensive regimens was associated with the improved CR and OS (22).

### HIV-Associated Burkitt Lymphoma

BL is a rare aggressive B cell NHL arising from the germinal center B cells (GCB) and is characterized by accelerated cellular growth and proliferation (23). BL accounts for 1-2% of B cell lymphomas in the general population, and up to 35% of HIV associated lymphomas (24–26). Patients with HIV-associated BL have higher incidence of extra-nodal involvement, B symptoms, advance stage and worse performance status that other patients with BL (27, 28). EBV is detected in 25-40% of HIV-BL patients compared to 3-5% in the general population, and has been suggested as a driver of lymphogenesis by some studies (29, 30).

Wang et al. (31) demonstrated that HIV-BL patients treated with the CODOX-M/IVAC regimen had similar CR and event-free survival (EFS) rates compared to HIV negative patients. Montoto et al. (32) reported a 3-year EFS and OS of 75% and 52%, respectively, in 30 HIV-BL patients treated with CODOX-M/IVAC regimen. Barnes et al. (33) retrospectively analyzed 80 BL patients at the Massachusetts General Hospital and the Dana-Farber Cancer Institute from 1992 to 2009, and 14 (18%) of them were HIV positive. 40 patients received rituximab in addition to chemotherapy (R-CODOX-M/R-IVAC), compared with 40 patients treated with CODOX-M/IVAC alone. The 3-year PFS and OS of 68% and 68%, respectively, in 14 HIV-BL patients, compared with 68% and 72, respectively, in 66 HIV negative BL patients. Barnes et al. (33) reported a 3 year OS of 77% in patients with BL when rituximab was added to CODOX-M/IVAC(R-CODOX-M/IVAC) chemotherapy regimen, compared to 66% on the group that was not treated with rituximab. Therefore, the R-CODOX-M/IVAC regimen could improve the OS compared with the CODOX-M/IVAC regimen in the HIV-positive BL patients.

The Hyper-CVAD/MA regimens were the Hyper-CVAD alternated with the MA. The Hyper-CVAD regimen consists of hyperfractionated cyclophosphamide, vincristine, doxorubicin and dexamethasone. The MA regime consists of methotrexate and cytarabine. Cortes J et al. (34) enrolled 13 patients with HIV-BL were treated with hyper-CVAD alternating with high-dose methotrexate and cytarabine for a total of eight cycles. 12 patients (92%) achieved a CR and one achieved a PR. The median survival was 12 months, with 48% of patients alive after 2 years.

Dunleavy et al. (35) enrolled 30 newly diagnosed BL patients from 2000 to 2009, and 11 were positive for HIV. HIV-BL underwent the SCEPOCH-RR chemotherapy regimen, which is lower-dose short-course combination with a double dose of rituximab. After a median follow-up of 73 months, 100% (72–100) of HIV-BL patients had freedom from progression of disease and overall survival was 90% (60–98).

Evens et al. (36) retrospectively analyzed 641 BL patients across 30 US cancer centers from 2009 to 2018, and 142 (22%) of them had co-existing HIV infection. Regarding therapy, 194 patients (30%) were treated with the R-CODOX-M/IVAC regimen, 195 patients (30%) were treated with R-hyper-CVAD/MA regimen, and 181 patients (28%) were treated with the DA-EPOCH-R regimen. Following these regimens, 455 patients (71%) achieved the CR, and 494 patients (77%) achieved the ORR among all newly treated BL patients. The ORRs were 86%, 79%, and 78% in the BL patients treated with the R-CODOX-M/IVAC, R-hyper-CVAD/MA, and DA-EPOCH-R, respectively. The CRs were 80%, 74%, and 71% in patients treated with the R-CODOX-M/IVAC, R-hyper-CVAD/MA, and DA-EPOCH-R, respectively. Treatment-related mortality (TRM) were 13% (n=18) and 9% (n=43) in the HIV-positive BL patient and in the HIV-negative patients, respectively (p=0.17). With a median follow-up of 45 months, 3-year PFS among all patients was 64% (95% CI 60%-68%) and OS was 70% (95% CI 65%-73%), respectively. Compared to chemotherapy alone, the addition of rituximab to the treatment of BL patients improved the 3 year PFS (67% vs. 38%, p<0.001) and OS (72% vs. 44%, p<0.001). A detailed analysis of HIV-BL in this BL cohort was reported by Alderuccio JP et al (37). separately. Alderuccio JP et al (37). retrospectively analyzed 249 HIV-BL patients. With a median follow up of 4.5 years, the 3-year PFS and OS were 61% (95% CI 55%-67%) and 66% (95%CI 59%-71%), respectively. Remarkably, HIV features no longer influence prognosis in contemporaneously treated HIV-BL. Other studies have suggested that high-intensity regimens may improve outcomes and increase cure rates of HIV-BL patients with well controlled HIV using cART, compared to lower-intesity chemotherapies (38–40).

### HIV-Associated HL

The HL was considered the most common type of non-AIDS-defining cancer in patients living with HIV. Powles et al. (41) demonstrated that the incidence of HL in patients living with HIV (PLWHIV) was increased by 5-20 times compared with the HIV-negative population. Before cART, the outcomes of the HIV-associated HL were poor. Now, with the wide use of cART and standard chemotherapy, the prognosis of these patients is similar to that of the general population.

The ABVD regimen consists of doxorubicin, bleomycin, vinblastine, and dacarbazine (42). Montoto et al. (43) retrospectively analyzed 224 newly diagnosed HL patients with the consecutive treatment with ABVD regimen in 5 hospitals in London from 1997 to 2010, of which 93 (41.5%) had HIV and were on cART. Compared with HIV-negative patients, the HIV-positive population had more advanced stage [III/IV; 74/93 (80%) vs. 45/131 (35%); p<0.001], higher International Prognostic Score [IPS 3-7; 63/93 (68%) vs. 34/131 (26%); p<0.001], more B symptoms [75/93 (81%) vs. 52/131 (40%); p<0.001], more bone marrow involvement [42/93 (45%) vs. 5/131 (4%); p<0.001], and lower lymphocyte count [median (×10^9^/L); 0.9 vs. 1.5; p<0.001]. The CR rates were 74% and 79% in the HIV-positive and HIV-negative patients, respectively (p=0.34). With a median follow-up of 60 months, the 5-year OS rates were 81% and 88% for the HIV-positive and HIV-negative HL patients, respectively (p=0.15). Meanwhile, the 5-year EFS rates were 59% and 66% in the HL patients with the HIV-positive or negative, respectively (p=0.5). The study demonstrated that the HIV infection does not adversely affect the survival of the HL patients when they have the ABVD chemotherapy regimen.

The BEACOPP regimen consists of bleomycin, etoposide, doxorubicin, cyclophosphamide, vincristine, oral procarbazine, and oral prednisone (42). Hentrich et al. (44) enrolled 108 newly diagnosed HIV-associated HL patients from 42 institutions in Germany and Austria from March 2004 to October 2010, of which 85 (79%) were on cART. The patients with the localized favorable stage (IA/B or IIA/B without risk factors) were treated with 2-4 cycles of the ABVD regimen followed by involved-field (IF) radiation (30 Gy). The patients with the localized unfavorable stage (IA/B or IIA/B with at least one risk factor) were treated with 4 cycles of the BEACOPP regimen or 4 cycles of the ABVD regimen plus IF radiation (30 Gy). In patients with the advanced stage (III/IV), 6-8 cycles of the BEACOPP regimen were administered. Of the 108 patients in the prospective, multicenter clinical trial study, 23 (21%) HL patients had an localized favorable stage, 14 (13%) HL patients had an localized unfavorable stage, and 71 (66%) HL patients had an advanced stage, and the CR rates were 96%, 100%, and 86%, respectively. The 2-year OS rates were 95.7%, 100%, and 86.8%, respectively. The survival rate had no significant difference between various groups. TRM for patients with localized favorable, localized unfavorable, and advanced disease was 4% (1/23), 0%, and 7% (5/70), respectively. Some other studies also demonstrated that the HIV infection did not adversely affect the prognosis of the HL patients (45, 46). The front line chemotherapy for HIV-associated lymphoma are summarized in **Table 1**.

**Table 1:** Front line chemotherapy for HIV-associated diffuse large B-cell lymphoma, Burkitt’s lymphoma and Hodgkin’s lymphoma in the cART era.

Chemotherapy regimen	Number of patients	Overall response rate	Complete response	Survival	Reference
**HIV-associated DLBCL**					
DA-EPOCH (retrospective)	39	87%	74%	OS 60% at 53 months	(17)
R-CHOP (retrospective)	97	86%	73%	5y-DFS 94% 5y-OS 78%	(18)
SC-EPOCH-RR (phase 2)	33	94%	91%	5y-PFS 84% 5y-OS 68%	(19)
R-CHOP (phase 3)	80	65.7%	57.6%	PFS 45weeks OS 139weeks at 137weeks	(20)
R-CDE (phase 2)	74	–	70%	2y-OS 64%	(21)
**HIV-associated BL**					
CODOX-M/IVAC (retrospective)	80	88%	–	3y-EFS 61-75% 3y-OS 52-66%	(31–33)
R-CODOX-M/IVAC (retrospective)	80	90%	–	3y-EFS 74% 3y-OS 77%	(33)
SC-EPOCH-RR (phase 2)	11	100%	100%	PFS 90% OS 100% at 73 months	(35)
DA-EPOCH-R (retrospective)	142	78%	71%	3y-PFS 67% 3y-OS 72%	(36)
**HIV-associated HL**					
ABVD (retrospective)	93	91%	74%	5y-EFS 59% 5y-OS 81%	(43)
ABVD/BEACOPP (phase 2)	localized favorable 23	–	96%	2y-OS 95.7%	(44)
	localized unfavorable 14		100%	2y-OS 100%	
	Advanced 71		86%	2y-OS 86.8%	
ABVD (retrospective)	68	–	–	2y-PFS 89% 2y-OS 94%	(46)

## Relapsed and Refractory ARL

The outcome of patients with ARL becomes very poor when the lymphoma relapsed or is refractory to the first-line chemotherapy. So far, there is no standard second-line therapy. Autologous hematopoetic stem cell transplant (AutoHCT) can be considered when the patients have remission as second-line therapy (47).

Alvarnas et al. (45) reported a 2-year PFS and OS of 79.8% and 82%, respectively, for patients with relapse ARL treated with modified carmustine, etoposide, cytarabine, and melphalan (BEAM), followed by AutoHCT. Bastos-Oreiro et al. (48) enrolled 33 patients with relapse/refractory ARL and 45 matched controls, who all underwent high-dose chemotherapy and AutoHCT from 2000 to 2016. With a median follow up of 70 months, the global OS was 71%. Patients with ARL had a OS of 64% compared to 75% in the HIV negative group (p=0.57).

The BMT CTN 0903/AMC 080 trial (49) represents the only prospective multi-center study of the use of allogeneic hematopoietic cell transplantation (alloHCT) in HIV-associated hematologic malignancies. 17 HIV-associated hematologic malignancies, which including 3 NHL and 1 HL, were enrolled in the clinical trial and underwent alloHCT. There was no non-relapse mortality (NRM) at 100 days. With a median follow-up of 24.4 months, the 1-year and 2-year NRM rate were 11.8% (95% CI, 1.8%-32.2%) and 18.3% (95% CI, 4.1%-40.7%), respectively. The 1-year and 2-year OS were 58.8% (95% CI, 32.5%-77.8%) and 52.3% (95% CI, 26.8%-72.7%), respectively. Similar data have been published from an international collaboration to investigate HIV cure by alloHCT (50). Among 23 HIV-associated hematologic malignancies undergoing alloHCT, 13 died within 2 years. Among patients who achieved complete donor chimerism, there was a profound long-term reduction in the HIV reservoir.

Chimeric antigen receptor T (CAR-T) cell therapy has shown efficacy in many hematologic malignancies, including acute lymphocytic leukemia (ALL) and DLBCL. However, CAR-T has been rarely used and results have been limited to case reports (51–53).

## Conclusions

Patients with ARL has significantly improved due to the wide use of cART. Using similar curative chemoimmunotherapy regimens as the ones given in patients with B cell NHL without HIV, has also improved these outcomes. More prospective randomized clinical trials with ARL patients are required in the future to facilitate the use of new drugs for the ARL.

## Author Contributions

CW wrote the essay. CW and YL revised the introduction and the first half of the article. JL helped to revise the framework of the article. All authors contributed to the article and approved the submitted version.

## Funding

The study was supported in part by the National Natural Science Foundation of China (grant 81670100).

## Conflict of Interest

The authors declare that the research was conducted in the absence of any commercial or financial relationships that could be construed as a potential conflict of interest.

## Publisher’s Note

All claims expressed in this article are solely those of the authors and do not necessarily represent those of their affiliated organizations, or those of the publisher, the editors and the reviewers. Any product that may be evaluated in this article, or claim that may be made by its manufacturer, is not guaranteed or endorsed by the publisher.

## References

[1] 1993 Revised Classification System for HIV Infection and Expanded Surveillance Case Definition for AIDS Among Adolescents and Adults. MMWR Recomm Rep (1992) 41(RR-17):1–19. 1361652

[2] Yarchoan R Uldrick TS. HIV-Associated Cancers and Related Diseases. N Engl J Med (2018) 378(22):2145. doi: 10.1056/NEJMc1804812 29539283 PMC6890231

[3] Simard EP Pfeiffer RM Engels EA. Cumulative Incidence of Cancer Among Individuals With Acquired Immunodeficiency Syndrome in the United States. Cancer (2011) 117(5):1089–96. doi: 10.1002/cncr.25547 PMC3052856 20960504

[4] Polesel J Clifford GM Rickenbach M Dal Maso L Battegay M Bouchardy C. Non-Hodgkin Lymphoma Incidence in the Swiss HIV Cohort Study Before and After Highly Active Antiretroviral Therapy. AIDS (2008) 22(2):301–6. doi: 10.1097/QAD.0b013e3282f2705d 18097233

[5] Sparano JA. HIV-Associated Lymphoma: The Evidence for Treating Aggressively But With Caution. Curr Opin Oncol (2007) 19(5):458–63. doi: 10.1097/CCO.0b013e3282c8c835 17762571

[6] Biancotto A Grivel JC Iglehart SJ Vanpouille C Lisco A Sieg SF. Abnormal Activation and Cytokine Spectra in Lymph Nodes of People Chronically Infected With HIV-1. Blood (2007) 109(10):4272–9. doi: 10.1182/blood-2006-11-055764 PMC1885500 17289812

[7] Meister A Hentrich M Wyen C Hübel K. Malignant Lymphoma in the HIV-Positive Patient. Eur J Haematol (2018) 101(1):119–26. doi: 10.1111/ejh.13082 29663523

[8] Rudresha AH Khandare PA Lokanatha D Linu AJ Suresh Babu MC Lokesh KN. HIV/AIDS-Related Lymphoma: Perspective From a Regional Cancer Center in India. Blood Res (2019) 54(3):181–8. doi: 10.5045/br.2019.54.3.181 PMC6779940 31730692

[9] Re A Cattaneo C Rossi G. Hiv and Lymphoma: From Epidemiology to Clinical Management. Mediterr J Hematol Infect Dis (2019) 11(1):e2019004. doi: 10.4084/mjhid.2019.004 30671210 PMC6328036

[10] Noy A. Optimizing Treatment of HIV-Associated Lymphoma. Blood (2019) 134(17):1385–94. doi: 10.1182/blood-2018-01-791400 PMC7493463 30992269

[11] Aboulafia DM. Non-Hodgkin Lymphoma in People With HIV. Lancet HIV (2019) 6(4):e209–10. doi: 10.1016/S2352-3018(19)30039-6 30826280

[12] Wu D Chen C Zhang M Wang S Shi J. The Clinical Features and Prognosis of 100 AIDS-Related Lymphoma Cases. Sci Rep (2019) 9(1):5381. doi: 10.1038/s41598-019-41869-9 30926889 PMC6441082

[13] Milligan MG Bigger E Abramson JS Sohani AR Zola M Kayembe MKA. Impact of HIV Infection on the Clinical Presentation and Survival of Non-Hodgkin Lymphoma: A Prospective Observational Study From Botswana. J Glob Oncol (2018) 4:1–11. doi: 10.1200/JGO.17.00084 PMC6223476 30241264

[14] Cingolani A Cozzi Lepri A Teofili L Galli L Mazzotta V Baldin GM. Survival and Predictors of Death in People With HIV-Associated Lymphoma Compared to Those With a Diagnosis of Lymphoma in General Population. PloS One (2017) 12(10):e0186549. doi: 10.1371/journal.pone.0186549 29088223 PMC5663375

[15] Wang C Xia B Ning Q Zhao H Yang H Zhao Z. High Prevalence of Hepatitis B Virus Infection in Patients With Aggressive B Cell Non-Hodgkin’s Lymphoma in China. Ann Hematol (2018) 97(3):453–7. doi: 10.1007/s00277-017-3188-2 29188315

[16] Schommers P Hentrich M Hoffmann C Gillor D Zoufaly A Jensen B. Survival of AIDS Related Diffuse Large B-Cell Lymphoma, Burkitt Lymphoma, and Plasmablastic Lymphoma in the German HIV Lymphoma Cohort. Br J Haematol (2015) 168(6):806–10. doi: 10.1111/bjh.13221 25403997

[17] Little RF Pittaluga S Grant N Steinberg SM Kavlick MF Mitsuya H. Highly Effective Treatment of Acquired Immunodeficiency Syndrome-Related Lymphoma With Dose-Adjusted EPOCH: Impact of Antiretroviral Therapy Suspension and Tumor Biology. Blood (2003) 101(12):4653–9. doi: 10.1182/blood-2002-11-3589 12609827

[18] Coutinho R Pria AD Gandhi S Bailey K Fields P Cwynarski K. HIV Status Does Not Impair the Outcome of Patients Diagnosed With Diffuse Large B-Cell Lymphoma Treated With R-CHOP in the cART Era. AIDS (2014) 28(5):689–97. doi: 10.1097/QAD.0000000000000133 24418826

[19] Dunleavy K Little RF Pittaluga S Grant N Wayne AS Carrasquillo JA. The Role of Tumor Histogenesis, FDG-PET, and Short-Course EPOCH With Dose-Dense Rituximab (SC-EPOCH-RR) in HIV-Associated Diffuse Large B-Cell Lymphoma. Blood (2010) 115(15):3017–24. doi: 10.1182/blood-2009-11-253039 PMC2858473 20130244

[20] Kaplan LD Lee JY Ambinder RF Sparano JA Cesarman E Chadburn A. Rituximab Does Not Improve Clinical Outcome in a Randomized Phase 3 Trial of CHOP With or Without Rituximab in Patients With HIV-Associated Non-Hodgkin Lymphoma: AIDS-Malignancies Consortium Trial 010. Blood (2005) 106(5):1538–43. doi: 10.1182/blood-2005-04-1437 PMC1895225 15914552

[21] Boue F Gabarre J Gisselbrecht C Reynes J Cheret A Bonnet F. Phase II Trial of CHOP Plus Rituximab in Patients With HIV-Associated Non-Hodgkin’s Lymphoma. J Clin Oncol (2006) 24(25):4123–8. doi: 10.1200/JCO.2005.05.4684 16896005

[22] Barta SK Xue X Wang D Tamari R Lee JY Mounier N. Treatment Factors Affecting Outcomes in HIV-Associated Non-Hodgkin Lymphomas: A Pooled Analysis of 1546 Patients. Blood (2013) 122(19):3251–62. doi: 10.1182/blood-2013-04-498964 PMC3821722 24014242

[23] Crombie J LaCasce A. The Treatment of Burkitt Lymphoma in Adults. Blood (2021) 137(6):743–50. doi: 10.1182/blood.2019004099 33171490

[24] Kimani SM Painschab MS Horner MJ Muchengeti M Fedoriw Y Shiels MS. Epidemiology of Haematological Malignancies in People Living With HIV. Lancet HIV (2020) 7(9):e641–51. doi: 10.1016/S2352-3018(20)30118-1 PMC10199168 32791045

[25] Gibson TM Morton LM Shiels MS Clarke CA Engels EA. Risk of Non-Hodgkin Lymphoma Subtypes in HIV-Infected People During the HAART Era: A Population-Based Study. AIDS (2014) 28(15):2313–8. doi: 10.1097/QAD.0000000000000428 PMC4260326 25111081

[26] Hernández-Ramírez RU Qin L Lin H Leyden W Neugebauer RS Althoff KN. Association of Immunosuppression and HIV Viraemia With Non-Hodgkin Lymphoma Risk Overall and by Subtype in People Living With HIV in Canada and the USA: A Multicentre Cohort Study. Lancet HIV (2019) 6(4):e240–9. doi: 10.1016/S2352-3018(18)30360-6 PMC6531288 30826282

[27] Davi F Delecluse HJ Guiet P Gabarre J Fayon A Gentilhomme O. Burkitt-Like Lymphomas in AIDS Patients: Characterization Within a Series of 103 Human Immunodeficiency Virus-Associated Non-Hodgkin’s Lymphomas. J Clin Oncol (1998) 16(12):3788–95. doi: 10.1200/JCO.1998.16.12.3788 9850023

[28] Han X Jemal A Hulland E Simard EP Nastoupil L Ward E. HIV Infection and Survival of Lymphoma Patients in the Era of Highly Active Antiretroviral Therapy. Cancer Epidemiol Biomarkers Prev (2017) 26(3):303–11. doi: 10.1158/1055-9965.EPI-16-0595 27756777

[29] Grande BM Gerhard DS Jiang A Griner NB Abramson JS Alexander TB. Genome-Wide Discovery of Somatic Coding and Noncoding Mutations in Pediatric Endemic and Sporadic Burkitt Lymphoma. Blood (2019) 133(12):1313–24. doi: 10.1182/blood-2018-09-871418 PMC6428665 30617194

[30] Panea RI Love CL Shingleton JR Reddy A Bailey JA Moormann AM. The Whole-Genome Landscape of Burkitt Lymphoma Subtypes. Blood (2019) 134(19):1598–607. doi: 10.1182/blood.2019001880 PMC6871305 31558468

[31] Wang ES Straus DJ Teruya-Feldstein J Qin J Portlock C Moskowitz C. Intensive Chemotherapy With Cyclophosphamide, Doxorubicin, High-Dose Methotrexate/Ifosfamide, Etoposide, and High-Dose Cytarabine (CODOX-M/IVAC) for Human Immunodeficiency Virus-Associated Burkitt Lymphoma. Cancer (2003) 98(6):1196–205. doi: 10.1002/cncr.11628 12973843

[32] Montoto S Wilson J Shaw K Heath M Wilson A McNamara C. Excellent Immunological Recovery Following CODOX-M/IVAC, an Effective Intensive Chemotherapy for HIV-Associated Burkitt’s Lymphoma. AIDS (2010) 24(6):851–6. doi: 10.1097/QAD.0b013e3283301578 20124971

[33] Barnes JA Lacasce AS Feng Y Toomey CE Neuberg D Michaelson JS. Evaluation of the Addition of Rituximab to CODOX-M/IVAC for Burkitt’s Lymphoma: A Retrospective Analysis. Ann Oncol (2011) 22(8):1859–64. doi: 10.1093/annonc/mdq677 21339382

[34] Cortes J Thomas D Rios A Koller C O'Brien S Jeha S. Hyperfractionated Cyclophosphamide, Vincristine, Doxorubicin, and Dexamethasone and Highly Active Antiretroviral Therapy for Patients With Acquired Immunodeficiency Syndrome-Related Burkitt Lymphoma/Leukemia. Cancer (2002) 94(5):1492–9. doi: 10.1002/cncr.10365 11920506

[35] Dunleavy K Pittaluga S Shovlin M Steinberg SM Cole D Grant C. Low-Intensity Therapy in Adults With Burkitt’s Lymphoma. N Engl J Med (2013) 369(20):1915–25. doi: 10.1056/NEJMoa1308392 PMC3901044 24224624

[36] Evens AM Danilov A Jagadeesh D Sperling A Kim SH Vaca R. Burkitt Lymphoma in the Modern Era: Real-World Outcomes and Prognostication Across 30 US Cancer Centers. Blood (2021) 137(3):374–86. doi: 10.1182/blood.2020006926 PMC8765121 32663292

[37] Alderuccio JP Olszewski AJ Evens AM Collins GP Danilov AV Bower M. HIV-Associated Burkitt Lymphoma: Outcomes From a US-UK Collaborative Analysis. Blood Adv (2021) 5(14):2852–62. doi: 10.1182/bloodadvances.2021004458 PMC8341354 34283175

[38] Hesseling PB Kouya F Katayi E Mbah G Wharin P. Burkitt’s Lymphoma: The Prevalence of HIV/AIDS and the Outcome of Treatment. S Afr Med J (2018) 108(2):84–5. doi: 10.7196/SAMJ.2017.v108i2.12441 29429436

[39] Noy A. HIV Lymphoma and Burkitts Lymphoma. Cancer J (2020) 26(3):260–8. doi: 10.1097/PPO.0000000000000448 PMC9302611 32496459

[40] Atallah-Yunes SA Murphy DJ Noy A. HIV-Associated Burkitt Lymphoma. Lancet Haematol (2020) 7(8):e594–600. doi: 10.1016/S2352-3026(20)30126-5 32735838

[41] Powles T Robinson D Stebbing J Shamash J Nelson M Gazzard B. Highly Active Antiretroviral Therapy and the Incidence of Non-AIDS-Defining Cancers in People With HIV Infection. J Clin Oncol (2009) 27(6):884–90. doi: 10.1200/JCO.2008.19.6626 19114688

[42] Eichenauer DA Aleman BMP André M Federico M Hutchings M Illidge T. Hodgkin Lymphoma: ESMO Clinical Practice Guidelines for Diagnosis, Treatment and Follow-Up. Ann Oncol (2018) 29(Suppl 4):iv19–29. doi: 10.1093/annonc/mdy080 29796651

[43] Montoto S Shaw K Okosun J Gandhi S Fields P Wilson A. HIV Status Does Not Influence Outcome in Patients With Classical Hodgkin Lymphoma Treated With Chemotherapy Using Doxorubicin, Bleomycin, Vinblastine, and Dacarbazine in the Highly Active Antiretroviral Therapy Era. J Clin Oncol (2012) 30(33):4111–6. doi: 10.1200/JCO.2011.41.4193 PMC5320889 23045581

[44] Hentrich M Berger M Wyen C Siehl J Rockstroh JK Müller M. Stage-Adapted Treatment of HIV-Associated Hodgkin Lymphoma: Results of a Prospective Multicenter Study. J Clin Oncol (2012) 30(33):4117–23. doi: 10.1200/JCO.2012.41.8137 23045592

[45] Alvarnas JC Le Rademacher J Wang Y Little RF Akpek G Ayala E. Autologous Hematopoietic Cell Transplantation for HIV-Related Lymphoma: Results of the BMT CTN 0803/AMC 071 Trial. Blood (2016) 128(8):1050–8. doi: 10.1182/blood-2015-08-664706 PMC5000843 27297790

[46] Besson C Lancar R Prevot S Brice P Meyohas MC Marchou B. High Risk Features Contrast With Favorable Outcomes in HIV-Associated Hodgkin Lymphoma in the Modern cART Era, ANRS CO16 LYMPHOVIR Cohort. Clin Infect Dis (2015) 61(9):1469–75. doi: 10.1093/cid/civ627 26223997

[47] Alencar AJ Moskowitz CH. Autologous Stem Cell Transplantation in the Management of Relapsed Non-Hodgkin Lymphoma. J Clin Oncol (2021) 39(5):467–75. doi: 10.1200/JCO.20.01751 33434059

[48] Bastos-Oreiro M Balsalobre P Miralles P Berenguer J Dorado N Bailen R. Autologous Stem Cell Transplantation for Lymphoma in HIV+ Patients: Higher Rate of Infections Compared With Non-HIV Lymphoma. Bone Marrow Transplant (2020) 55(9):1716–25. doi: 10.1038/s41409-020-0846-0 32132653

[49] Ambinder RF Wu J Logan B Durand CM Shields R Popat UR. Allogeneic Hematopoietic Cell Transplant for HIV Patients With Hematologic Malignancies: The BMT CTN-0903/AMC-080 Trial. Biol Blood Marrow Transplant (2019) 25(11):2160–6. doi: 10.1016/j.bbmt.2019.06.033 PMC6907401 31279752

[50] Salgado M Kwon M Gálvez C Badiola J Nijhuis M Bandera A. Mechanisms That Contribute to a Profound Reduction of the HIV-1 Reservoir After Allogeneic Stem Cell Transplant. Ann Intern Med (2018) 169(10):674–83. doi: 10.7326/M18-0759 30326031

[51] Allred J Bharucha K Özütemiz C He F Janakiram M Maakaron J. Chimeric Antigen Receptor T-Cell Therapy for HIV-Associated Diffuse Large B-Cell Lymphoma: Case Report and Management Recommendations. Bone Marrow Transplant (2021) 56(3):679–82. doi: 10.1038/s41409-020-01018-7 PMC7956159 32764581

[52] Abramson JS Irwin KE Frigault MJ Dietrich J McGree B Jordan JT. Successful Anti-CD19 CAR T-Cell Therapy in HIV-Infected Patients With Refractory High-Grade B-Cell Lymphoma. Cancer (2019) 125(21):3692–8. doi: 10.1002/cncr.32411 31503324

[53] Abbasi A Peeke S Shah N Mustafa J Khatun F Lombardo A. Axicabtagene Ciloleucel CD19 CAR-T Cell Therapy Results in High Rates of Systemic and Neurologic Remissions in Ten Patients With Refractory Large B Cell Lymphoma Including Two With HIV and Viral Hepatitis. J Hematol Oncol (2020) 13(1):1. doi: 10.1186/s13045-019-0838-y 31900191 PMC6942268

